# Bendamustine-based therapy as first-line treatment for non-Hodgkin lymphoma

**DOI:** 10.1007/s12032-014-0944-1

**Published:** 2014-04-22

**Authors:** Lidia Gil, Maciej Kazmierczak, Renata Kroll-Balcerzak, Mieczyslaw Komarnicki

**Affiliations:** Department of Haematology and Bone Marrow Transplantation, Poznan University of Medical Sciences, 84 Szamarzewski Street, 60-569 Poznan, Poland

**Keywords:** Bendamustine, First-line treatment, Non-Hodgkin lymphoma, Indolent lymphoma, Mantle cell lymphoma, Aggressive lymphoma

## Abstract

Recently, bendamustine has become an important agent in the treatment for patients with lymphoid malignancies. Although the drug has received approval for second-line therapy in indolent lymphoma, a growing body of evidence suggests its efficacy and safety in first-line use. The results of randomised and observational studies with bendamustine as front-line therapy in non-Hodgkin lymphoma (NHL) with emphasis on efficacy and toxicity are presented. Furthermore, completed and ongoing clinical trials evaluating upfront bendamustine effectiveness in combination with other agents are discussed. The review refers mainly to indolent lymphoma, mantle cell lymphoma and aggressive lymphoma, as the most commonly diagnosed NHL types. Finally, we elaborated on the safety profile of bendamustine and the perspectives of using the drug as a first-line therapy.

## Introduction

Bendamustine is an anticancer drug, which has recently evolved as an important agent for a number of lymphoid malignancies in Europe and the USA.

The drug consists of an alkylating nitrogen mustard group bound to a purine-like benzimidazole ring, and because of this unique bifunctional structure the bendamustine activity profile is significantly different from classical alkylators. Although the precise mechanism of action has not been elucidated yet, it is known that bendamustine induces DNA cross-linking and DNA breaks and induces cell death by apoptosis through intrinsic and extrinsic pathways, which in turn may deregulate the cell cycle and lead to a “mitotic” catastrophe [1]. Preclinical studies and clinical observations suggest that bendamustine has limited cross-resistance with other alkylating agents and demonstrates significant synergism with anti-CD20 monoclonal antibody rituximab and purine analogues [2, 3].

Based on two multicentre randomised studies, bendamustine has received approval for second-line therapy in relapsed/refractory indolent non-Hodgkin lymphoma (NHL) [4, 5]. A growing body of evidence suggests good efficacy and acceptable tolerability of bendamustine as the first-line option for indolent lymphoma, mantle cell lymphoma (MCL) and selected patients with aggressive lymphoma.

This review presents data for bendamustine use in first-line therapy of NHL, taken from all available relevant articles published in the years 2006–2013, supplemented with abstracts from recent haematology and oncology scientific meetings. A summary of available data, source publications and conference abstracts is presented in Tables 1 and 2.

**Table 1:** Bendamustine in first-line treatment for indolent or mantle cell lymphoma—prospective studies

Study	Patients (*n*)	Diagnosis	ORR %	CR %	PFS	Ref
BOP versus COP prospective phase III Herold 2006	164	FL, LPL, MCL	66 versus 76	22 versus 20	5 yrs: 59 % versus 46 %	8
BR versus CHOPR prospective phase III Rummel 2013 StiL study	549	FL, MCL, LPL, MZL, SLL	93 versus 91	40 versus 30	69.5 versus 31.2 months	9
BR versus CVPR or CHOPR prospective phase III Flinn 2012 BRIGHT study	436	Indolent, MCL	94 versus 84	31 versus 25		10
BR prospective phase II Luminari 2013	69	MZL, SLL, LPL	84	58	1 yrs: 90 %	14
BR + cytarabine prospective phase II Visco 2013	20	MCL	100	95	2 yrs: 95 %	19
RiBVD (BR + bortezomib + dexamethasone) prospective phase II Gressin 2013 LYSA study	76 elderly	MCL	87	60		22
BR + lenalidomide prospective phase I–II Jerkeman 2013	51	MCL	97	79	Not reached in 18-month follow-up	21
BR + R maintenance prospective phase II Rummel 2012	162	LPL	86			9, 12
BR prospective phase II Salar 2012	60	MALT	100	98		13
B + ofatumumab prospective phase II Fowler 2012	49	Indolent	98	60		17
BR + mitoxantrone prospective phase II Boccomini 2012	76 elderly	FL	96	75		16
BR + bortezomib prospective phase II Flinn 2012	55	Indolent	89	47		15
B + ofatumumab + dexamethasone prospective II phase Magni 2012	19 elderly	MCL	94	89		20

*B;* bendamustine, *BR;* bendamustine + rituximab, *BOP;* bendamustine + vincristine + prednisone, *COP;* cyclophosphamide + vincristine + prednisone, *CVPR;* cyclophosphamide + vincristine + prednisone + rituximab, *CHOPR;* cyclophosphamide + doxorubicin + vincristine + prednisone + rituximab, *RiBVD;* rituximab + bendamustine + bortezomib + dexamethasone, *FL;* follicular lymphoma, *LPL;* lymphoplasmacytic lymphoma, *MCL;* mantle cell lymphoma, *MZL;* marginal zone lymphoma, *SLL;* small lymphocytic lymphoma, *MALT;* mucosa-associated lymphoid tissue, *ORR;* overall response rate, *CR;* complete remission, *PFS;* progression-free survival, *Ref;* reference, *yrs;* years

**Table 2:** Bendamustine in first-line treatment for aggressive lymphoma

Study	Patients (n), diagnosis	ORR %	CR %	PFS months	OS months	Ref
BR, retrospective Horn 2012	20, DLBCL, elderly	55	20	8.3	19.4	25
BR, retrospective Kuntz 2010	15, DLBCL	46	33	14.1	23.1	27
BR, retrospective Hammersen 2013	21 DLBCL MCL	91	14	8	24	28
BR, retrospective Walter 2012	15, DLBCL	62	38	6	9	26
BR, prospective phase II Weidmann 2011	14, DLBCL, MCL elderly	69	54	7.7	7.7	24
BR prospective phase II Park 2013	23, DLBCL, elderly	93	60		9.9	29

*BR;* bendamustine + rituximab, *DLBCL;* diffuse large B-cell lymphoma, *MCL;* mantle cell lymphoma, *ORR;* overall response rate, *CR;* complete remission, *PFS;* progression-free survival, *OS;* overall survival, *Ref;* reference

## Indolent lymphoma

Low-grade lymphoma, considered as an incurable disease, represents about 40 % of all NHLs. Rituximab in combination with chemotherapy, usually a CHOP (cyclophosphamide, doxorubicin, vincristine, prednisone) regimen, is a standard first-line treatment, especially in follicular lymphoma subset [6]. Encouraging results of bendamustine in relapsed or refractory indolent NHL have drawn attention to the first-line use of the drug in this setting. German Haematology Outpatient Centres have reported a continuous increase in the use of bendamustine in combination with rituximab (BR) as upfront therapy in patients with indolent NHL, despite no European Medicines Agency (EMA) approval for this indication [7].

The first randomised study evaluating the effectiveness of bendamustine in treatment-naive indolent lymphoma patients was published in 2006 [8]. The drug (bendamustine), in combination with vincristine and prednisone (BOP), was compared with a COP (cyclophosphamide, vincristine, prednisone) regimen in 164 patients with follicular lymphoma, MCL and lymphoplasmacytic lymphoma (LPL). There were no statistically significant differences between groups with respect to overall response rate (ORR) (BOP vs. COP: 66 vs. 76 %; *p* = 0.1) and overall survival (BOP vs. COP: 61 vs. 46 %; *p* = 0.2). The median time to progression was significantly longer in BOP responders (84 months) than in those who responded to the COP regimen (28 months), *p* = 0.0369, which translated into the probability of 5-year progression-free survival (PFS) as 59 % versus 46 %, respectively. Additionally, both haematological and non-haematological complications were less common and less severe in the BOP group than in the COP group (Table 3).

**Table 3:** Haematological and non-haematological toxicity of bendamustine-based regimens

Study	Patients diagnosis	Haematological toxicity % grade 3–4		Non-haematological toxicity % grade 3–4		Ref
BOP versus COP prospective phase III Herold 2006	164	Anaemia	10 versus 13	Alopecia	4 versus 48	8
	FL, LPL	Thrombocytopenia	19 versus 34	Vomiting	0 versus 1	
	MCL	Leucopenia	4 versus 1	Neuropathy	1 versus 0	
BR versus CHOPR prospective phase III Rummel 2013 StiL study	549	Anaemia	3 versus 5	Alopecia*	0 versus 100	9
	FL, LPL	Leucopenia	37 versus 72	Neuropathy*	7 versus 29	
	MZL, SLL, MCL	Thrombocytopenia	5 versus 6	Infection*	37 versus 50	
BR versus CVPR or CHOPR prospective phase III Flinn 2012 BRIGHT study	436	Anaemia*	5 versus 5	Alopecia*	4 versus 51	10
	Indolent	Leucopenia*	39 versus 87	Vomiting*	29 versus 13	
	MCL	Thrombocytopenia*	10 versus 12	Neuropathy*	14 versus 44	
				Infection*	55 versus 57	
RiBVD prospective phase II Gressin 2013	76	Anaemia	2	Fatigue	5	22
	MCL	Neutropenia	21	Diarrhoea	8	
	Elderly	Thrombocytopenia	15	Neuropathy	4	

* All grades

More recently, an important phase III study comparing bendamustine with rituximab (BR) to CHOPR (cyclophosphamide, doxorubicine, vincristine, prednisone, rituximab) regimen was presented [9]. Following the treatment for 549 patients with indolent or MCL, ORR was similar for BR and CHOPR groups (93 vs. 91 %), complete remission (CR) was significantly increased in the BR group (40 vs. 30 %, *p* = 0.021) and PFS was significantly longer in the BR group (69.5 vs. 31.2 months, *p* < 0.0001). There were fewer toxic complications after BR therapy.

Another randomised study compared results of BR therapy with CVPR (cyclophosphamide, vincristine, prednisone, rituximab) or CHOPR in the treatment for 436 patients with indolent NHL or MCL [10, 11]. ORR was 94 % after BR in comparison with 84 % after CVPR or CHOPR regimen, and CR rate was higher for the BR group (31 vs. 25 %, *p* = 0.0225). Analysis indicated that adverse reactions were reported for both BR and CVPR or CHOPR therapy (Table 3). Subgroup analysis for indolent lymphoma (MCL excluded), revealed similar results with respect to CR rate for BR and CVPR or CHOPR regimens (28 vs. 25 %).

BR is effective in the treatment for patients with LPL, as was seen in the StiL study and confirmed by Rummel in a phase II trial with 86 % of ORR, where BR therapy was followed by rituximab maintenance therapy [9, 12]. Another subgroup of NHL patients with mucosa-associated lymphoid tissue (MALT) lymphoma, treated in a Spanish study with BR, achieved 98 % CR rate, and overall response was observed in 100 % [13].

A number of ongoing and recently finished clinical trials investigating bendamustine in combination with other drugs (mitoxantrone, lenalidomide, bortezomib) reveal promising ORR and CR in the indolent lymphoma setting [13–17]. These results translate into prolonged progression-free survival, which today is considered a treatment success in low-grade NHL patients.

Bendamustine has not yet been approved as a first-line therapy of NHL in Europe, but as the preliminary reports on its use in treatment-naïve patients are encouraging, there are an increasing number of centres using bendamustine as a compassionate drug [7, 18]. A retrospective multicentre analysis of the Spanish Registry of indolent NHL patients revealed high effectiveness, with an ORR of 95 % and CR of 66.5 %, and favourable tolerance profile of BR regimen in newly diagnosed indolent lymphoma patients.

## Mantle cell lymphoma

Mantle cell lymphoma has poorer prognosis and outcome than other subtypes of NHL. Young and fit patients are usually treated with intensive therapy followed by autologous stem cell transplantation. A growing body of data indicates a high response rate after bendamustine in patients with MCL, not only in rescue therapy but also as upfront treatment. In a phase II study with bendamustine in combination with cytarabine and rituximab in untreated MCL patients, ORR was 100 % and 2-year PFS was 95 % [19]. Subgroup analysis of prospective, randomised studies has also shown superiority of BR regimen in comparison to CHOPR-like treatment [9]. The CR rate in the BRIGHT study was 27 % for BR versus 50 % for CHOPR or CVPR regimen, and PFS (progression-free survival) in the StiL study for BR was 35.4 months versus 22.1 months for CHOPR (*p* = 0.0044).

Ongoing clinical studies are evaluating bendamustine in combination with lenalidomide, ibrutinib, temsirolimus or ofatumumab in MCL therapy [20–22]. Preliminary results from the LYSA trial, including 76 elderly patients newly diagnosed with MCL, indicated that 4 cycles of rituximab, bendamustine, bortezomib, dexamethasone (RiVBD) yielded 87 % ORR rate and 60 % CR rate [22]. Fifty-one elderly patients (>65 years) with previously untreated MCL grade II–IV were provided with BR combined with lenalidomide. Following six cycles of the treatment, the ORR was 97 %, CR was 79 % and OS after 2 years amounted to 87 % [21].

The results of clinical studies published thus far, assessing the use of bendamustine in upfront treatment for MCL, seem to indicate a risk-stratified approach, based on the MCL international prognostic index (MIPI). Bendamustine in combination with rituximab could be recommended as a first-line therapy in elderly MCL patients with high or intermediate MIPI risk [23].

## Aggressive lymphoma

The combination of rituximab and cyclophosphamide, doxorubicin, vincristine and prednisone is the standard first-line treatment regimen in patients with diffuse large B-cell lymphoma (DLBCL); however, the available data do not apply to elderly or frail patients. Only a few smaller studies have investigated the role of bendamustine in this setting.

A phase II study demonstrated a CR rate of 54 % in elderly patients treated with BR as first-line therapy with a good safety profile [24]. Retrospective studies confirmed the efficacy of the BR regimen in unfit DLBCL patients. Results are acceptable and manageable for toxicity; however, they seem to be generally unacceptable for PFS and overall survival [25–27]. Defining prognostic factors as GCB-subtype of DLBCL might predict a better outcome in bendamustine treated patients [28]. Performance and comorbidity assessment have a great impact on the outcome of frail patients with aggressive lymphoma [29].

## Bendamustine safety profile

The clinical studies published so far have reported fairly low or mild toxicity of bendamustine-containing regimens [30]. The adverse events (AE) observed in lymphoma patients treated with bendamustine are summarised in Table 3. In general, bendamustine seems to present a favourable toxicity profile, and the most common complications involve haematological events such as anaemia, leucopenia, neutropenia or thrombocytopenia. Despite being reversible, these toxicities are common, with as much as a 94 % occurrence rate for all grade events. The most frequently reported non-haematological toxicities related to bendamustine treatment include nausea, infections, fatigue, constipation, diarrhoea, headache and vomiting. A multicentre phase III clinical trial comparing first-line BR versus CHOPR revealed a much lower rate of serious AE in the BR arm than in CHOPR treated patients (49 vs. 74) [9]. Moreover, a granulocyte colony-stimulating factor (G-CSF) was used only in 4 % of the patients receiving BR and in 20 % of those receiving CHOPR treatment. Similarly, non-haematological AEs were of lower grade or incidence in the BR group than in the CHOPR arm. A meta-analysis of randomised, controlled trials comparing the bendamustine-containing regimen to any other regimen demonstrated no effect of bendamustine on the rate of infection when compared to either alkylating agents or fludarabine in haematological, as well as in solid, malignancies despite, remarkably, lymphopenia [31]. A few case studies were published reporting on the safety and effectiveness of the BR regimen in DLBCL patients with severe liver impairment [32]. Low toxicity of bendamustine, even in these unfavourable settings, makes the drug safe and effective in special patient populations.

The exact mechanism behind bendamustine’s low toxicity remains not fully understood, and additional basic research aimed at its elucidation is required. It also seems advisable to assess long-term bendamustine toxicity and its potential interactions with other second-line treatments.

Increasing interest in bendamustine as an upfront therapy has prompted a cost-effectiveness analysis of this drug. When compared to CHOPR or CVPR regimens, the BR was found to be cost-effective, and due to its more favourable toxicity profile it incurred lower costs related to AE management [33].

## Perspectives

The remarkably low toxicity profile and high efficacy of bendamustine provide a basis for the use of this drug in the treatment for a variety of lymphoma subsets. Recent preclinical data and clinical studies have confirmed the activity of bendamustine in heavily pretreated patients with Hodgkin lymphoma or peripheral T-cell lymphoma [34]. The efficacy of bendamustine has been evaluated in treatment for multiple myeloma, with encouraging results [35, 36]. These impressive observations may lead to the design of prospective trials evaluating bendamustine in upfront therapy in selected patients in these settings.

It was demonstrated that bendamustine provided clinical benefits when combined with other agents, i.e. monoclonal antibodies, purine analogues or more modern drugs such as lenalidomide, bortezomib, ibrutinib or idelalisib. There are a number of clinical trials, expected to be completed in 2014 and 2015, that are assessing the dose-limited toxicity and maximum tolerated dose of bendamustine in combination with those and other drugs. Such observations create new possibilities in the therapy of lymphoid malignancies.

## References

[1] Leoni LM, Bailey B, Reifert J, Bendall HH, Zeller RW, Corbeil J, Elliott G, Niemeyer CC (2008). Bendamustine (Treanda) displays a distinct pattern of cytotoxicity and unique mechanistic features compared with other alkylating agents. Clin Cancer Res.

[2] Gandhi V (2002). Metabolism and mechanisms of action of bendamustine: rationales for combination therapies. Semin Oncol.

[3] Chow KU, Boehrer S, Geduldig K, Krapohl A, Hoelzer D, Mitrou PS, Weidmann E (2001). In vitro induction of apoptosis of neoplastic cells in low-grade non-Hodgkin’s lymphomas using combinations of established cytotoxic drugs with bendamustine. Haematologica.

[4] Kahl BS, Bartlett NL, Leonard JP, Chen L, Ganjoo K, Williams ME, Czuczman MS, Robinson KS, Joyce R, van der Jagt RH, Cheson BD (2010). Bendamustine is effective therapy in patients with rituximab-refractory, indolent B-cell non-Hodgkin lymphoma: results from a multicenter study. Cancer.

[5] Friedberg JW, Cohen P, Chen L, Robinson KS, Forero-Torres A, La Casce AS, Fayad LE, Bessudo A, Camacho ES, Williams ME, van der Jagt RH, Oliver JW, Cheson BD (2008). Bendamustine in patients with rituximab-refractory indolent and transformed non-Hodgkin’s lymphoma: results from a phase II multicenter, single-agent study. J Clin Oncol.

[6] Kahl B (2008). Chemotherapy combinations with monoclonal antibodies in non-Hodgkin’s lymphoma. Semin Hematol.

[7] Knauf WU, Abenhardt W, Nusch A, Grugel A, Marschner N. Bendamustine-rituximab (BR) replaces R-CHOP as “standard of care” in the treatment of indolent non-Hodgkin lymphoma in German Hematology Outpatient Centers. Blood. 2012; 120: Abstract 3666.

[8] Herold M, Schulze A, Niederwieser D, Franke A, Fricke HJ, Richter P, Freund M, Ismer B, Dachselt K, Boewer C, Schirmer V, Weniger J, Pasold R, Winkelmann C, Klinkenstein C, Schulze M, Arzberger H, Bremer K, Hahnfeld S, Schwarzer A, Muller C (2006). Bendamustine, vincristine and prednisone (BOP) versus cyclophosphamide, vincristine and prednisone (COP) in advanced indolent non-Hodgkin’s lymphoma and mantle cell lymphoma: results of a randomised phase III trial (OSHO# 19). J Cancer Res Clin Oncol.

[9] Rummel MJ, Niederle N, Maschmeyer G, Banat GA, von Grunhagen U, Losem C, Kofahl-Krause D, Heil G, Welslau M, Balser C, Kaiser U, Weidmann E, Durk H, Ballo H, Stauch M, Roller F, Barth J, Hoelzer D, Hinke A, Brugger W (2013). Bendamustine plus rituximab versus CHOP plus rituximab as first-line treatment for patients with indolent and mantle-cell lymphomas: an open-label, multicentre, randomised, phase 3 non-inferiority trial. Lancet.

[10] Flinn IW, Richard H, Van der Jagt, Kahl B. An open-label, randomized study of bendamustine and rituximab (BR) compared with rituximab, cyclophosphamide, vincristine, and prednisone (R-CVP) or rituximab, cyclophosphamide, doxorubicin, vincristine, and prednisone (R-CHOP) in first-line treatment of patients with advanced indolent non-Hodgkin’s lymphoma (NHL) or mantle cell lymphoma (MCL). The bright study. Blood. 2012; 120: Abstract 902.

[11] van der Jagt R (2013). Bendamustine for indolent non-Hodgkin lymphoma in the front-line or relapsed setting: a review of pharmacokinetics and clinical trial outcomes. Expert Rev Hematol.

[12] Rummel MJ, Lerchenmuller C, Greil R, Gorner M, Hensel M, Engel E, Jaeger U, Breuer F (2012). Bendamustin-rituximab induction followed by observation or rituximab maintenance for newly diagnosed patients with waldenstrom macroglobulinemia: results from prospective, randomized, multicenter study (StiL NHL 7-2008—MAINTAIN). Blood.

[13] Salar A, Doningo-Domenech E, Panizo C, Nicolas C, Bargay J, Muntanola A, Canales M. Final results of a multicenter phase II trial with bendamustine and rituximab as first line treatment for patients with MALT lymphoma. Blood. 2012;120:Abstract 3691.

[14] Luminari S, Goldaniga MC, Cesaretti M, Orsucci L, Tucci A, Pulsoni A, Salvi F. Bendamustine and rituximab combination in untreated indolent non follicular B-cell non-Hodgkin’s lymphoma. A phase II study from the Fondazione Italiana Linfomi. Blood. 2013;122:Abstract 3049.

[15] Flinn IW, Thompson DS, Boccia RV, Miletello G, Lipman A, Flora D, Cuevas D. Bendamustine, bortezomib, and rituximab, in patients with previously untreated low grade lymphoma: A phase II Trial of the Sarah Cannon Research Institute. Blood. 2012;120:Abstract 1624.

[16] Boccomini C, Ladetto M, Rigacci L, Arcaini L, Ceccarelli M, Lobetti-Bodoni C, Volpetti S. Breif chemoimmunotherapy rituximab, bendamustine, mitoxantrone (R-BM) Followed by RITUXIMAB Consolidation in elderly patients with untreated advanced stage follicular lymphoma (FL): preliminary results of prospective phase II study by Fondazione Italiana Linfomi. Blood. 2012;120:Abstract 2720.

[17] Fowler NH, Kahanic SP, Forero A, Munteanu MC, Davis GL, Brown P. Results of a Phase II Study with Bendamustine and Ofatumumab in Untreated Indolent B-cell Non-Hodgkin’s Lymphoma. Blood. 2011;118:Abstract 778. 10.1007/s00277-014-2269-8 25630297

[18] Martinez-Chamorro C, Pinizo C, Quero C, Deben G, Paz J, Batlle A, Serrano A (2013). Bendamustine in combination with rituximab as first-line treatment for indolent non-Hodgkin lymphoma: retrospective analysis of a Spanish Registry. Blood.

[19] Visco C, Finotto S, Zambello R, Paolini R, Menin A, Zanotti R, Zaja F, Semenzato G, Pizzolo G, D’Amore ES, Rodeghiero F (2013). Combination of rituximab, bendamustine, and cytarabine for patients with mantle-cell non-Hodgkin lymphoma ineligible for intensive regimens or autologous transplantation. J Clin Oncol.

[20] Magni M, Di Nicola M, Carlo-Stella C, Devizzi L, Guidetti A, Matteucci P. Safety, tolerability and activity of ofatumumab, bendamustine and dexamethasone combination as first-line treatment of mante-cell lymphoma in the elderly: a multicenter study. Blood. 2011;118:Abstract 1647.

[21] Jerkeman M, Albertsson-Lindblad A, Kolstad A, Laurell A, Raty R, Grrnbck K. Lenalidomide, bendamustine, and rituximab as firs-line therapy for patients > 65 years with mantle cell lymphoma: preliminary results from the nordic lymphoma group MCL4 Phase I-II Trial. Blood. 2013;122:Abstract 4377.

[22] Gressin R, Callanan M, Daguindau N, Tempescul A, Carras S, Cartron G. The ribvd regimen (rituximab IV, bendamustine IV, velcade SC, dexamethasone IV) offers a high complete response rate in elderly patients with untreated mantle cell lymphoma. Preliminary results of the Lysa Trial. Blood. 2013;122:Abstract 370.

[23] Vose JM (2013). Mantle cell lymphoma: 2012 update on diagnosis, risk-stratification, and clinical management. Am J Hematol.

[24] Weidmann E, Neumann A, Fauth F, Atmaca A, Al-Batran SE, Pauligk C, Jager E (2011). Phase II study of bendamustine in combination with rituximab as first-line treatment in patients 80 years or older with aggressive B-cell lymphomas. Ann Oncol.

[25] Horn J, Kleber M, Hieke S, Schmitt-Graff A, Wasch R, Engelhardt M (2012). Treatment option of bendamustine in combination with rituximab in elderly and frail patients with aggressive B-non-Hodgkin lymphoma: rational, efficacy, and tolerance. Ann Hematol.

[26] Walter E, Schmitt T, Dietrich S, Ho A, Witzens-Harig M (2013). Rituximab and bendamustine in patients with CD20 + diffuse large B-cell lymphoma not eligible for cyclophosphamide, doxorubicin, vincristine and prednisone-like chemotherapy. Leuk Lymphoma.

[27] Kuntz E, Schmitt T, Dietrich S, Bonn S, Ho A, Witzens-Harig M. Rituximab and bendamustine in patients with diffuse large B-cell lymphoma not eligible for CHOP like chemotherapy. Onkologie. 2010;33:Abstract P173. 10.3109/10428194.2012.682311 22475324

[28] Hammarsen J, Sommer M, Gossel C, Teichgraber U, Hahnfeld S, Scholl S, Fricke HJ. Safety and efficacy od bendamustine in patients with agressive non-hodgkin lymphomas (NHL). Blood. 2013;122:Abstract 4371.

[29] Park S, Richards KL, Asch A, Olajide O, Deal AM, Ivanova A. A multicenter phase II study of bendamustine in combination with rituximab in older patients with previously untreated diffuse large B-cell lymphoma. Blood. 2013;122:Abstract 1791. 10.1111/bjh.14232 PMC5063684 27448091

[30] Tageja N (2013). Bendamustine: safety and efficacy in the management of indolent non-hodgkins lymphoma. Clin Med Insights Oncol.

[31] Gafter-Gvili A, Gurion R, Rannani P, Shpilberg O, Vidal L. Bendamustine is not associated with an increase in infections—systematic review and meta-analysis of randomized controlled trials. Blood. 2013;122:Abstract 5125. 10.1002/hon.2350 27734524

[32] McCloskey JK, Broome CM, Cheson BD (2013). Safe and effective treatment of aggressive non-hodgkin lymphoma with rituximab and bendamustine in patients with severe liver impairment. Clin Adv Hematol Oncol.

[33] Dewilde S, Woods B, Castaigne JG, Parker C, Dunlop W (2014). Bendamustine-rituximab: a cost-utility analysis in first-line treatment of indolent non-Hodgkin’s lymphoma in England and Wales. J Med Econ.

[34] Damaj G, Gressin R, Bouabdallah K, Cartron G, Choufi B, Gyan E, Banos A, Jaccard A, Park S, Tournilhac O, Schiano de Collela JM, Voillat L, Joly B, Le Gouill S, Saad A, Cony-Makhoul P, Vilque JP, Sanhes L, Schmidt-Tanguy A, Bubenheim M, Houot R, Diouf M, Marolleau JP, Bene MC, Martin A, Lamy T (2013). Results from a prospective, open-label, phase II trial of bendamustine in refractory or relapsed T-cell lymphomas: the BENTLY trial. J Clin Oncol.

[35] Ponisch W, Mitrou PS, Merkle K, Herold M, Assmann M, Wilhelm G, Dachselt K, Richter P, Schirmer V, Schulze A, Subert R, Harksel B, Grobe N, Stelzer E, Schulze M, Bittrich A, Freund M, Pasold R, Friedrich T, Helbig W, Niederwieser D (2006). Treatment of bendamustine and prednisone in patients with newly diagnosed multiple myeloma results in superior complete response rate, prolonged time to treatment failure and improved quality of life compared to treatment with melphalan and prednisone—a randomized phase III study of the East German study group of hematology and oncology (OSHO). J Cancer Res Clin Oncol.

[36] Berenson JR, Yellin O, Bessudo A, Boccia RV, Noga SJ, Gravenor DS, Patel-Donnelly D, Siegel RS, Kewalramani T, Gorak EJ, Nassir Y, Swift RA, Mayo D (2013). Phase I/II trial assessing bendamustine plus bortezomib combination therapy for the treatment of patients with relapsed or refractory multiple myeloma. Br J Haematol.

